# The assessment of the spondyloarthritis international society concept and criteria for the classification of axial spondyloarthritis and peripheral spondyloarthritis: A critical appraisal for the pediatric rheumatologist

**DOI:** 10.1186/1546-0096-10-14

**Published:** 2012-05-31

**Authors:** Ruben Burgos-Vargas

**Affiliations:** 1 Department of Rheumatology, Hospital General de México, Faculty of Medicine, Universidad Nacional Autónoma de México, Dr. Balmis 148, Colonia Doctores, México, DF 06720, Mexico

**Keywords:** Spondyloarthritis, Ankylosing spondylitis, Juvenile SpA, Juvenile AS, Enthesitis related arthritis, ASAS criteria

## Abstract

This review refers to the origin and current state of the assessment of the SpondyloArthritis International Society (ASAS) criteria for the classification of axial and peripheral spondyloarthritis (SpA) and the possible implications in the pediatric population. The ASAS criteria evolved from the idea that the earlier the recognition of patients with ankylosing spondylitis, the better the efficacy of tumor necrosis factor blockers. Strategies included the development of new concepts, definitions, and techniques for the study of clinical signs and symptoms. Of relevance, the new definition of inflammatory back pain (IBP) and the introduction of sacroiliitis by magnetic resonance imaging represented the most important advance in the early identification of AS in the “pre-radiographic stage” of the disease. AS is considered in this paper as a disease continuum with symptoms depending on age at onset. The application of those specific strategies in children and adolescents with SpA seems limited because the most important manifestation in the early stage of disease is not IBP, but peripheral arthritis and enthesitis. In this instance, the logical approach to juvenile onset SpA according to ASAS criteria should not be through the axial criteria but rather the peripheral set of criteria.

## Review

Ankylosing spondylitis (AS) is the prototype of a group of arthritis conditions collectively called SpA1, which includes undifferentiated SpA (u-SpA), reactive arthritis (ReA), and subsets of psoriatic arthritis (PsA), Crohn’s disease, and ulcerative colitis. The characteristics of these conditions include axial and peripheral enthesitis and arthritis, certain extrarticular manifestations, family aggregation, and HLA-B27 association. The spectrum of SpA spans from early, undifferentiated forms to well-defined diseases such as AS. Despite the fact that u-SpA might be the initial stage of any definite form of SpA, mainly AS, a number of patients may have only a short and mild course of symptoms followed by complete remission of inflammation and no permanent consequences.

In contrast to this mild, self-limited disease, AS is the result of a long-standing process that combines chronic inflammation and new bone formation, mainly at the tendon and ligament attachments to bones. AS is usually characterized by inflammatory back pain (IBP) and morning stiffness, progressive reduction of the spinal mobility, lower-limb joint and entheses involvement, anterior uveitis, and non-specific inflammatory bowel disease (IBD) [1]. The prevalence of AS in the population is linked to that of HLA-B27 and occurs most frequently in HLA-B27 young males [2].

The diagnosis of AS is usually made eight to ten years after the onset of complaints (mostly IBP) and depends on the presence of axial signs and symptoms and radiographic changes of the sacroiliac joints (Table 1) [3]. The course of the disease varies from one individual to another. Disease activity may show a fluctuating pattern and the structural damage, particularly late spinal changes such as syndesmophyte formation and the notorious “bamboo spine”, that illustrates the relatively slow progression. With such variation in disease course, the long-term consequences of AS, particularly health related quality of life (HRQoL) and functioning, can differ among the individuals that suffer from the disease [4-6]. Finally, some data suggest that the mortality of AS is increased when compared to that of the general population [7,8].

**Table 1:** **Modified New York criteria for ankylosing spondylitis ref.**[3]

A. Diagnosis*		
1. Clinical criteria		
a)		Low back pain and stiffness for more tan three months, which improves by exercise, but is not relieved by rest
b)		Limitation of motion of the lumbar spine in both the sagittal and frontal planes
c)		Limitation of chest expansion relative to normal values correlated for age and sex
2. Radiological criterion:		
Sacroiliitis grade ≥2 bilaterally or grade 3–4 unilaterally		
**B. Grading**		
1.	Definite ankylosing spondylitis is considered if the radiologic criteria is associated with at least one clinical criterion	
2.	Probable ankylosing spondylitis if:	
	a)	Three clinical criteria are present
	b)	The radiologic criterion is present without any signs or symptoms satisfying the clinical criteria (other causes of sacroiliitis should be considered)
Radiographic criteria		
Grade 0 = normal		
Grade 1 = suspicious changes		
Grade 2 = minimal abnormality – small localized areas with erosions or sclerosis, without alteration in the joint width		
Grade 4 = severe abnormality –total ankylosis.		

*The modified New York criteria for ankylosing spondylitis are mostly used for classification.

All three clinical and the radiographic criteria refer exclusively to axial involvement, including the spinal, costovertebral, costosternal, and sacroiliac joints.

The proportion of children and adolescents that fulfill those criteria before they reach the age 17 years is probably <15%. In such a case they usually have a combination of peripheral and axial symptoms.

It is assumed that stated otherwise, publications on ankylosing spondylitis never refer to definite or probable disease, but to definite ankylosing spondylitis.

The term “ankylosing spondylitis” means “stiff vertebrae” (from the Greek ankylos and spondylos). Alternative names, most in disuse, include seronegative polyarthritis, seronegative spondarthritis, seronegative spondyloarthritis, seronegative spondylarthropathies, spondyloarthritides. The stereotypic AS patient has a long-standing and severe disease characterized by spinal deformity and vertebral ankylosis for which no effective therapy has been available. Yet, it is clear that not all patients with AS fit into that stereotype. Moreover, the introduction of tumor necrosis factor (TNF) blockers has resulted in the control of signs and symptoms related to inflammation and the improvement of most outcome measures, including HRQoL, particularly in patients with a short disease duration.

Within the last ten years, clinicians have tried to recognize and diagnose AS in the early inflammatory stage of the process that ultimately leads to bone proliferation [9,10]. The purpose of this effort was to treat AS in the early pre-radiographic stage of the disease with TNF-blockers and prevent its long-term consequences. These strategies were utilized to identify the early inflammatory stage of the disease in patients by focusing on the definition, classification, and diagnostic criteria of IBP and SpA, including the use of magnetic resonance imaging (MRI) for the detection of sacroiliac and vertebral inflammation, as well as the use of HLA-B27 testing. Ultimately, the Assessment of SpondyloArthritis International Society (ASAS) developed concept and classification criteria for axial SpA [11] and then for peripheral SpA [12]. At the same time, we learned about the efficacy of TNF blockers in controlling inflammation, but probably not in suppressing bone proliferation.

In this paper, the characteristics and development of the ASAS criteria for the classification of axial and peripheral SpA are reviewed. These classification criteria and their possible role in the classification of children and adolescents with SpA are examined by defining and comparing the juvenile and adult criteria as they apply to pre-radiographic AS, AS, u-SpA, and ASAS axial and peripheral SpA.

### The relationship between adult and juvenile-onset AS and SpA

Although the most common age at onset of AS is around 25 years, a variable percentage of patients start complaining in childhood or adolescence. Despite the fact that juvenile and adult onset forms differ in their mode of presentation, both forms represent a disease continuum in which age-related factors might play a role in clinical expression. Compared with juvenile onset AS, patients with adult onset disease have a higher prevalence of axial symptoms and, in contrast, a much lower prevalence of peripheral enthesitis and arthritis in the initial years of the disease [13-22]. Inconsistent genetic and minor synovial histologic differences between adults and juvenile patients have been also described [23-26].

The consequences of AS seem more severe in juvenile patients [13,15-17,19-22]. Despite the fact that there are no studies comparing other SpA in adult and childhood subjects, the information available up to now on SpA-related, u-SpA, and ReA does not reveal major differences between the two age-at-onset populations. No important differences in pathogenesis or therapeutics have been reported. Thus, the concept that juvenile and adult onset AS represent the same disease has important implications in the clinical and therapeutic field. Indeed, this concept does not consider the subgroup of PsA in younger children (mostly girls, with dactylitis, polyarthritis, and antinuclear antibodies).

In keeping with the traditional idea of Moll and Wright about the SpA group [27], we conceptualize juvenile-onset SpA, including AS, as a group of HLA-B27–associated pediatric rheumatic diseases characterized by enthesitis and arthritis involving in most cases the lower extremities in the initial years and, in a variable proportion of cases, the sacroiliac and spinal joints some years later [28]. This concept differs from that of the enthesitis related arthritis (ERA) and PsA subgroups of juvenile idiopathic arthritis (JIA) classification of the International League for Associations of Rheumatology (ILAR) [29]. In that classification, the overlapping of diagnostic manifestations between two or more subgroups excludes definite diagnosis and puts any such case in the “undifferentiated arthritis” category.

### The relationship between u-SpA and AS

**Figure 1:**
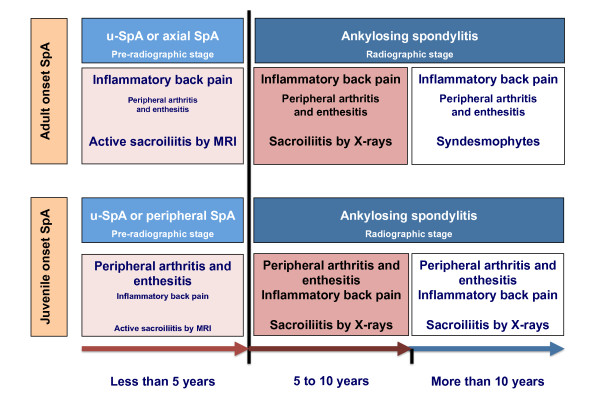
**The modified schematic representation of Rudwaleit’s scheme**[49]**on the transition of undifferentiated juvenile-onset spondyloarthritis (u-SpA) to ankylosing spondylitis (AS) in the context of axial and peripheral SpA.** Note: In this model, we present the idea that axial SpA is mostly representative of adult-onset patients (upper panel) whereas the subset that could mostly be adapted to children and adolescents with juvenile-onset SpA is that of peripheral SpA (lower panel). Time to transition is an estimation based on scarce published reports. Note that during the interval between disease onset and the 5th year of symptoms, the most frequent and characteristic symptoms and signs in juvenile-onset SpA are peripheral arthritis and enthesitis and not inflammatory back pain (small fonts; IBP). In contrast, IBP, and not peripheral symptoms, (small fonts) is the most important characteristic in adult patients. Beyond the 5th year of disease, the proportion of patients with both juvenile and adult onset SpA fulfilling the modified New York criteria for AS [3] increases to reach a maximum around 10 years after onset. Imaging of the sacroiliac joints, particularly the early inflammatory stage by magnetic resonance imaging (MRI) is essential in the study of adult patients with IBP. This approach does not seem the logical in the study of juvenile-onset SpA since IBP occurs in less than 15% in the initial years of disease. While the demonstration of edema in the sacroiliac joints by MRI has therapeutic implications in adult-onset SpA –the earliest the treatment, the better the response, the indications for early treatment of children and adolescents with SpA with tumor necrosis factor (TNF) blockers could mostly rely on peripheral symptoms. Regarding the ostechondral proliferative stage of spinal involvement, it is only probable that in juvenile-onset patient this stage takes a long time to develop, perhaps similar to that seen in adults.

The rationale for the development of the ASAS axial and peripheral classification criteria was to facilitate the recognition and detection of patients with back pain at risk of AS. It is widely known that most patients with early AS present with undifferentiated manifestations, most characteristically with IBP, less frequently with peripheral arthritis and enthesitis, and rarely with extrarticular manifestations. Thus, the initial stage of AS corresponds to that of u-SpA, a group that accounts for an important proportion of SpA in the community [30,31], specialized clinics [32], and multiplex-case families [33,34]. The initial descriptions of u-SpA dated back to 1983 and 1984 [35,36] and consisted of patients fulfilling the Amor, et al. [37] as well as the European Spondylarthropathy Study Group (ESSG) [38] classification criteria for SpA, but not fulfilling criteria for AS or the specific diagnostic features of ReA, PsA, Crohn’s disease, and ulcerative colitis. Besides IBP, peripheral arthritis and enthesitis have been prominent features at onset in patients with u-SpA [39,40]. In retrospect, most patients with u-SpA fulfill AS criteria within five to ten years after onset (Figure 1) [41-49].

Peripheral u-SpA has a childhood counterpart, which is traceable to clinical descriptions embraced under terms such as “probable Still’s disease” [50], type II (HLA-B27 + boys) oligoarticular juvenile rheumatoid arthritis (JRA) [51], atypical SpA [52], and most clearly to HLA-B27-associated SpA and enthesopathy in children [53] as well as the seronegative enthesopathy and arthropathy (SEA) syndrome [54]. This progression of juvenile-onset u-SpA to AS starts in most cases with peripheral symptoms and follows a clinical course leading to AS within ten years of disease [55-61] (Figure 1).

**Figure 2:**
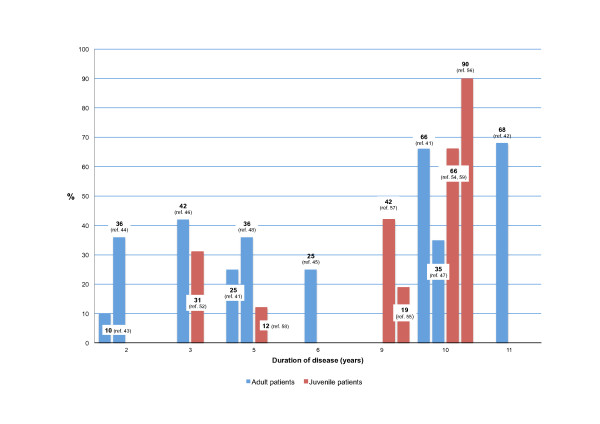
**Percentage of adult and juvenile onset patients with undifferentiated SpA (u-SpA) progressing to ankylosing spondylitis (AS) throughout the course of the disease as reported in retrospective studies.** Note: Variations are probably related to the characteristics of the population included in the study, entry criteria, and the type of assessments carried out. Interestingly, most references related to adult-onset patients include a moderately high proportion of patients with peripheral arthritis in combination or not with axial symptoms. Most studies consider AS the outcome measure, but some others referred to other parameters, for example radiographic sacroiliitis.

There are important variations in the proportion of adult-onset and juvenile-onset patients with u-SpA fulfilling AS criteria that occur throughout the disease courses in various studies (Figure 2). The reasons for these variations are probably related to the characteristics of the population included in the study, patient entry and inclusion criteria, design of the study, as well as the type and periodicity of clinical assessments. Information on those who remain in the undifferentiated stage or on those evolving into other SpA (i.e.: PsA) as well as on those entering into sustained remission, is limited in these studies.

In adults with u-SpA, progression to AS has been inconsistently associated with several factors. These include uveitis [41,46,48], HLA-B27 [41,43], alternate gluteal pain [43], peripheral arthritis [44], a high erythrocyte sedimentation rate [41], an elevated C- reactive protein (CRP) [46], recurrent oligoarthritis [45], and a low-grade radiographic sacroiliitis [46]. AS risk factors include low-grade radiographic sacroiliitis [46,62] and uveitis [46], and buttock pain [47].

In contrast, in children with JIA or JRA, radiographic sacroiliitis and AS associate in univariate or multivariate analyses with a somewhat different set of factors at different intervals such as HLA-B27 [54,61], DRB1*04 [61,63], age at onset of symptoms, male sex, and family history of AS [61], arthritis [54], polyarthritis [64], enthesitis [60,61], tarsitis [60], hip involvement [61,63], axial involvement [60-64], and psoriasis [61] (Table 2).

**Table 2:** **Variables associated with the likelihood of developing ankylosing spondylitis after a mean of 12.2 years (10 to14) ref. [**[60]]* and radiographic sacroiliitis after a mean of 14.9 years (11.7 to 25.1) ref. [[61]**]**

	Univariate analysis			Multlivariate analysis		
	OR	95%CI	p	OR	95%CI	p
Male	5.37	2.00-14.44	0.001	2.92	0.93-9.12	0.066
Onset age ≥8 years	21.12	2.809-159.48	0.003	9.21	1.11-76.40	0.039
Family history of AS	9.94	3.67-26.92	0.000	ND		
HLA-B27	10.35	3.63-29.54	0.000	5.29	1.63-17.17	0.005
DRB1*04	4.38	1.72-11.13	0.002	1.55	0.50-4.89	0.448
Enthesitis at 6 months*	42.5	6.3-1766.6	0.000	ND		
Enthesitis at 6 months	8.06	1.31-46.96	0.020	ND		
Enthesitis at 12 months*	22.1	6.1-118.1	0.000	ND		
Tarsitis at 6 months*	39.2	5.7-1641.1	0.000	ND		
Tarsitis at 12 months*	8.0	3.1-22.1	0.000	ND		
Hip arthritis at 6 months	6.34	2.30-17.46	0.000	4.98	1.32-18.70	0.017
IBP/SIJ* pain at 6 months	10.5	1.1-505.5	0.01	ND		
IBP at 6 months	16.22	2.16-121.9	0.007	ND		
Psoriasis	7.23	1.72-30.49	0.007	ND		

*35 patients with juvenile-onset ankylosing spondylitis compared with 75 patients with systemic, polyarticular, and pauciarthritis type 1 juvenile rheumatoid arthritis. OR and 95%CI were recalculated and differ from those reported in the original publication ref. [60]. In this sample, no multivariate analysis was performed.

314 patients with juvenile idiopathic arthritis ref. [61].

In a 15-year extended follow-up of 55 patients with enthesitis related arthritis years, DRB1*04 (OR 4.13, 95%CI 1.26-13.46, p = 0.19; univariate analysis), hip arthritis at 6 months (OR 46.73, 95%CI 1.16-39.34; multivariate analysis), and high sedimentation rate for at least 6 month (OR 4.09, 95%CI 1.05-15.91; multivariate analysis) were associated with radiographic sacroiliitis ref. [63].

OR = odds ratio. 95%CI = 95% confidence intervals. AS = ankylosing spondylitis. ND = not determined. IBP = inflammatory back pain. SIJ = sacroiliac joint pain.

Prospective data of patients with axial SpA according to ASAS criteria or IBP differ in some important aspects from retrospective studies. These differences are seen in the prospective German Spondyloarthritis Inception Cohort (GESPIC) [65], the Maastricht’s early SpA clinic population (ESPAC) [66], as well as the Leeds [67], and French (Devenir des Spondylarthropathies Indifférenciées Récentes or DESIR) [68] IBP clinics.

GESPIC [65] consisted of 462 patients with axial SpA, including 236 with AS of whom 50.4% had already developed AS within five years of symptoms. In these patients, male sex was predictive of radiographic sacroiliitis and >1 syndesmophyte and CRP ≤6 mg/L for >1 syndesmophyte and >1 bridging syndesmophyte. In ESPAC [66], 14 patients (21%) out of 68 with IBP had already developed AS within <2 years of symptoms, 24% had PsA and 15% each had IBD and uveitis. In the Leeds clinic data [67], 13 patients out of 40 (33%) with IBP had AS within two years and the others had PsA SpA (n = 3), ReA SpA (n = 6), IBD SpA (n = 1), and u-SpA (n = 17).

The combination of severe sacroiliitis on MRI and HLA-B27 was highly predictive for AS. The DESIR [68] study included 708 adults with IBP of <3 years disease duration. Of this group, 184 (26%) had AS, 77% SpA, and 67% axial SpA. HLA-B27 positivity was associated with a younger age at the onset of IBP, less delay in diagnosis, lower frequency of psoriasis, and higher frequencies of sacroiliitis and spondylitis on imaging.

The importance of such prospective studies is that up to one-third of patients with AS fulfill the modified New York criteria for AS within two years of symptoms and 50% by five years. While cohort studies include patients with IBP, those studies in retrospect include a significant number of patients with peripheral symptoms. Despite a trend for male sex, HLA-B27, and uveitis in patients with AS, most features of this peripheral AS disease do not differ from those of patients with axial SpA. Ultimately, these prospective risk factors include some factors already identified in retrospective studies and help define and illustrate the relationships between u-SpA, pre-radiographic AS, and axial and peripheral SpA.

Although Zeidler, et al. [49] proposed four possible outcomes for u-SpA, only two of them are clearly found in the rheumatology clinic: 1) a subgroup of patients representing the early stage of a definite, well-categorized SpA (for example, AS) and 2) a subgroup consisted of patients with “definite” u-SpA. The stage between the onset of symptoms and the demonstration of radiographic sacroiliitis in patients with u-SpA is the pre-radiographic stage of AS. This recent ability to recognize u-SpA patients in this stage, thereby allowing the earlier use of TNF blockers such as etanercept or infliximab, has appeared to result in better clinical responses [69]; this improvement has had a major influence in the development of the concept of axial SpA [9]. The initial strategy was the identification of patients with axial symptoms at risk of AS [9-11]; lately, the a new strategy has evolved in focusing on identification of those with peripheral disease at risk of axial SpA [12].

Rudwaleit, et al. [9] first calculated the pre-test probability of axial SpA and AS among mostly adult patients with any kind of back pain according to the sensitivity, specificity, and positive likelihood ratio (LR) of SpA features as appeared in different publications. He built two algorithms to be used in clinical practice, one starting with the identification of IBP in patients with back pain and the other with by HLA-B27 testing alone. The former algorithm increases the probability of having axial SpA (including AS) up to around 90% and the latter up to 59%.

Based on real-world clinical findings, Heuft-Dorenbosch, et al. [66] proposed changes on the level for placing MRI and HLA-B27 in the algorithm. In another study, IBP, HLA-B27, and sacroiliitis by MRI performed well in detecting axial SpA in patients referred by orthopedists and primary care physicians who had back pain >3 months, and age at onset of <45 years [70]. Ultimately, such criteria and algorithms provide the clinician, particularly the general practitioner and the orthopedic surgeon, with diagnostic strategies to differentiate IBP from mechanical back pain.

There is no such analyses yet in juvenile-onset SpA, but the association of some variables with the development of AS and radiographic sacroiliitis suggest a role for genetic, demographic, and clinical features for the progression of u-SpA to AS (Table 2). Essential in interpreting such information is the recognition that while the development of axial SpA starts with back pain, data on juvenile-onset SpA is derived from JIA and JRA data, conditions whose principal characteristic is not axial disease, but peripheral arthritis.

### Axial and peripheral ASAS SpA criteria

Today, the concept of axial SpA has moved from the non-radiographic sacroiliitis stage of AS to the wider spectrum of SpA, including the axial and peripheral categories [11,12] (Table 3). The ASAS criteria for SpA scope focus on the two most frequent groups of clinical features: the IBP group and the peripheral arthritis, enthesitis, and dactylitis group. In the initial studies of adult patients with IBP of less than 2 years, these diagnostic and classificatory properties of both the ASAS axial and peripheral SpA criteria appear to be better than those of reported by Amor, et al. [37] and ESSG [38] groups and implementation is under way. Drug efficacy studies are being carried out in patients with axial SpA to determine the role of TNF blockers in remission and prevention of structural damage [71-73].

**Table 3:** Axial and peripheral spondyloarthritis Assessment of Spondyloarthritis International Society classification criteria

Axial spondyloarthritis ref. [11]		Peripheral spondyloarthritis ref. [12]	
Individuals <45 years with back pain >3 months*		Individuals with arthritis or enthesitis or dactylitis	
Imaging	HLA-B27		
Sacroiliitis on MR or X-rays	Positive test		
Additional features needed for classification§			
At least one	At least two	At least one	At least two
Inflammatory back pain		Uveitis	Arthritis
Arthritis		Psoriasis	Enthesitis (heel pain)
Enthesitis (heel pain)		Crohn’s/colitis	Dactylitis
Uveitis		Previous infection	Inflammatory back pain (ever)
Dactylitis		HLA-B27	Family history for SpA
Psoriasis		Sacroiliitis on imaging	
Crohn’s/colitis			
Good response to non-steroidal antinflammatory drugs			
Family history for spondyloarthritis			
HLA-B27			
Elevated C reactive protein			

Classification of individuals as Axial SpA: *Requires two eligibility criteria (age at onset and duration of back pain) followed by two different approaches, one through MR and another through HLA-B27 testing. The former requires at least one and the latter at least two additional criteria.

Classification of individuals as peripheral SpA: Requires at least of three eligibility criteria (arthritis or enthesitis or dactylitis) plus one or two items depending on their diagnostic value.

§Despite axial and peripheral SpA share most additional criteria for classification, the definition of some individual criteria might be different. Please check such definitions in tables.

MR = Magnetic Resonance.

Nevertheless, there are more issues in axial [11] and peripheral [12] SpA criteria that need to be considered:

1) The existence of two sets of criteria has academic and research implications, yet their validation in various populations and clinical scenarios are needed before they would be widely used in clinical practice;

2) The cost of MRI and HLA-B27 testing may limit the applicability of ASAS criteria in countries with budget restrictions or in segments of the population not covered by any health security system;

3) Despite the fact that the definition of some variables listed in both axial and peripheral SpA criteria differ from each other to avoid classification overlaps, the existence of different definitions may be confusing (Table 4);

4) Except for radiographic sacroiliitis, signs and symptoms in the ASAS criteria for SpA refer to active inflammatory and not structural damage; and

5) Regarding nomenclature, the term “peripheral SpA” may appear to be contradictory in itself and confusing

**Table 4:** Definitions of parameters applied in the Assessment of Spondyloarthritis International Society classification criteria for axial and peripheral spondyloarthritis

	Axial SpA ref. [11]	Peripheral SpA ref. [12]
IBP	According to experts (14): ≥4 out of 5 parameters present: (1) age at onset 40 years; (2) insidious onset; (3) improvement with exercise; (4) no improvement with rest; (5) pain at night (with improvement upon getting up)	IBP in the past according to the rheumatologist’s judgment.
		In patients with current IBP (and concomitant peripheral manifestations), the ASAS classification criteria for axial SpA should be applied
Arthritis	Past or present active synovitis diagnosed by a physician	Current peripheral arthritis compatible with SpA (usually asymmetric and/or predominant involvement of the lower limbs), diagnosed clinically by a doctor
Enthesitis	Heel enthesitis: past or present spontaneous pain or tenderness at examination of the site of the insertion of the Achilles tendon or plantar fascia at the calcaneus.	Enthesitis: past or present spontaneous pain or tenderness at examination of an entheses.
		Any site of enthesitis can be affected whereas in the ASAS classification criteria for axial SpA only enthesitis of the heel is considered.
Uveitis	Past or present uveitis anterior, confirmed by an ophthalmologist	
Dactylitis	Past or present dactylitis, diagnosed by a physician	
Psoriasis	Past or present psoriasis, diagnosed by a physician	
IBD	Past or present Crohn’s disease or ulcerative colitis diagnosed by a physician	
Good response to NSAIDs	24–48 h after a full dose of a NSAID the back pain is not present any more or is much better	Not mentioned
Family history of SpA	Presence in first-degree (mother, father, sisters, brothers, children) or second-degree (maternal and paternal grandparents, aunts, uncles, nieces and nephews) relatives of any of the following: (1) AS; (2) psoriasis; (3) acute uveitis; (4) reactive arthritis; (5) IBD	
Elevated CRP	CRP concentration above upper normal limit in the presence of back pain, after exclusion of other causes for elevated CRP concentration.	Not mentioned
HLA-B27	Positive testing according to standard laboratory techniques	
Radiographic SI	Bilateral grade 2–4 or unilateral grade 3–4 sacroiliitis on plain radiographs, according to the modified New York criteria ref. [3]	
SI by MRI	Active inflammatory lesions of sacroiliac joints with definite bone marrow edema/osteitis, suggestive of sacroiliitis associated with SpA	
Preceding infection	NA	Urethritis/cervicitis or diarrhoea within 1 month before the onset of arthritis/enthesitis/dactylitis

SpA = spondyloarthritis. IBP = inflammatory back pain. ASAS = Assessment of SpondyloArthritis international Society. IBD = Inflammatory bowel disease. NSAIDs = non-steroidal anti-inflammatory drug. AS = ankylosing spondylitis. IBD = inflammatory bowel disease. CRP = C-reactive protein. MRI = magnetic resonance imaging.

### The role of axial and peripheral SpA criteria in children and adolescents

ASAS criteria for axial and particularly peripheral SpA may have important implications for the recognition of children and adolescents with SpA and the understanding of the relationship between juvenile and adult SpA. Children and adults would be classified under the same criteria, the long-term follow-ups of children with SpA would be more easily carried out, and the results of clinical trials and management would be interchangeable to some extent.

### Where do this leave us?

**Figure 3:**
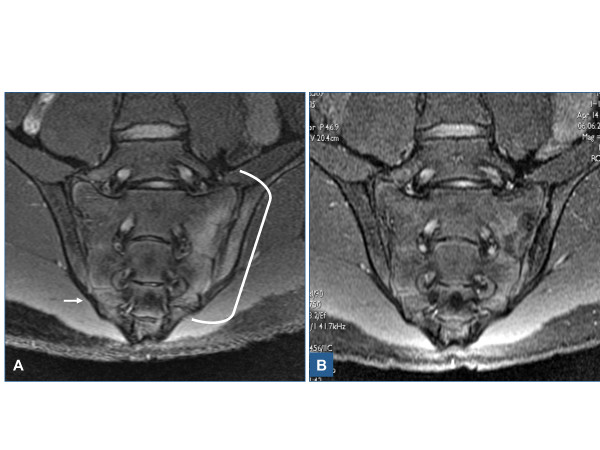
**TNF blocker effect on sacrolliitis.** Short Tau (t) (inversion time) Inversion Recovery (STIR) magnetic resonance (MR) imaging of the sacroiliac joints of a 16-year-old boy a 3-month history of gluteal pain and a 3-year history of peripheral arthritis and enthesitis before (A) and 14 weeks after 5 mg/kg infliximab treatment at baseline and weeks 2 and 6. In **(A)**, there is ample edema of the iliac bone and sacrum (line) and part of the sacrum inferior quadrant on the opposite side (arrow) that cleared-up after treatment **(B)**.

**Figure 4:**
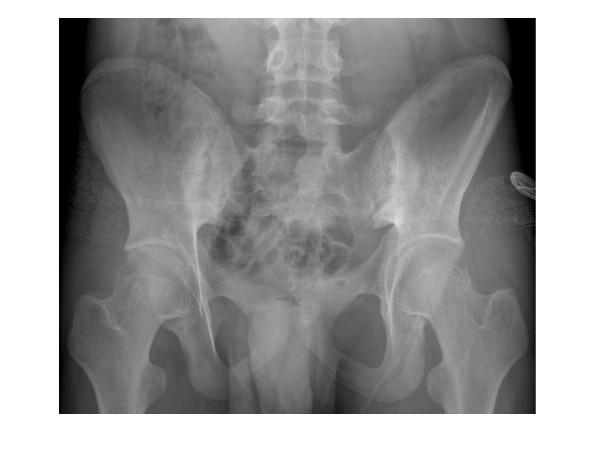
**Grade 3 bilateral sacroiliitis in a 14-year-old boy with 6 years disease duration.** There is subchondral sclerosis of the iliac bone, joint surface irregularities, which include some erosions on both sides, and joint space narrowing of the hips (From Burgos-Vargas, R. 2006, The juvenile-onset spondyloarthritides. In: Weisman MH, van der Heijde D, Reveille JD. Ankylosing spondylitis and the Spondyloarthropathies. Mosby. Philadelphia. pp 94–106).

**Figure 5:**
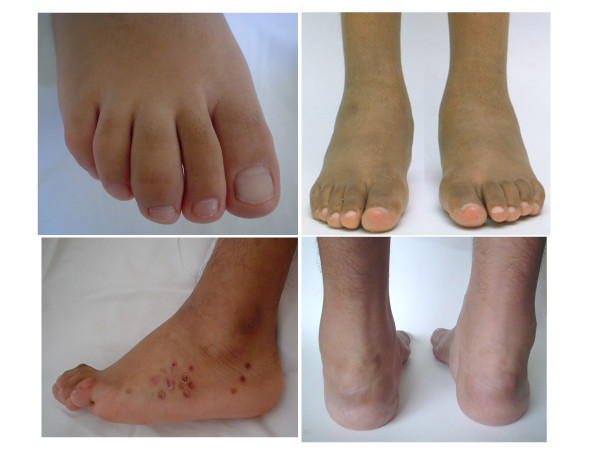
**Involvement of the feet in patients with SpA. A.** Dactylitis of the third digit in a 12-year-old boy with undifferentiated SpA. **B.** Diffuse swelling of the tarsal region of a 11- year-old boy with juvenile-onset AS. **C.** Tarsal swelling, hyperextension of the digits, and keratoderma blenorrhagica spots in a 16-year-old boy with chronic reactive arthritis. **D.** Diffuse swelling of the posterior aspect of feet including the ankles, Achilles, peroneal and tibial posterior tendons in a 12 year-old-boy with undifferentiated SpA (Modified from Burgos-Vargas R, Vázquez-Mellado J. Reactive arthritides. In: Cassidy JT, Petty RE (eds): Textbook of Pediatric Rheumatology. 5th Edition. Philadelphia. Elsevier Saunders. 2005:604–612 and Burgos-Vargas, R. 2006, The juvenile-onset spondyloarthritides. In: Weisman MH, van der Heijde D, Reveille JD. Ankylosing spondylitis and the Spondyloarthropathies. Mosby. Philadelphia. pp 94–106).

**Figure 6:**
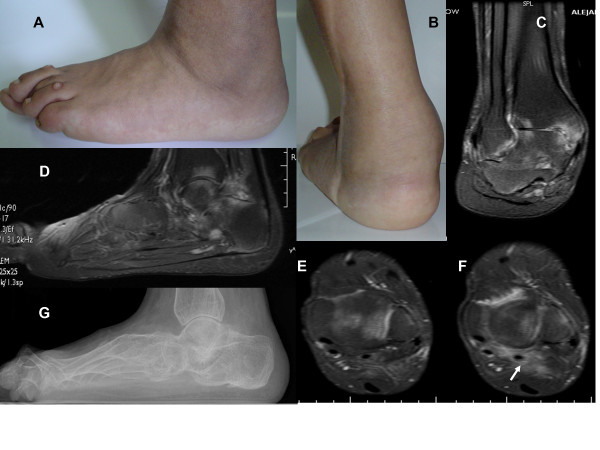
**Composite images of ankylosing tarsitis in a 16-year-old boy with AS of 9 year’s disease duration and complete ankylosis of the tarsal bones and grade 2 bilateral sacroiliitis. A** and **B.** Flat foot and swelling around the ankle. **C**, **D**, **E**, and **F**. T2-weighted-fat suppressed MR imaging showing edema in various tarsal bones, joint spaces (**C** and **D**), and soft tissues (**E** and **F**) surrounding the tendons of the posterior aspect of the foot on the coronal view (arrows) fat. **G.** Complete ankylosis of the tarsal bones and an enthesophyte at the plantar fascia attachment (modified from: Burgos-Vargas R. A case of childhood-onset ankylosing spondylitis: diagnosis and treatment. Nat Clin Pract Rheumatol 2009;5:52–7).

Before quickly accepting these ASAS criteria for children and adolescents, however, it is important to answer two specific questions:

1) Is there a rationale for alternative criteria for children with ERA, PsA, and undifferentiated arthritis according to ILAR criteria? ERA and PsA (and perhaps some cases of undifferentiated arthritis) are the subgroups representing juvenile-onset SpA in the ILAR JIA classification criteria for JIA [29]. The concept of ERA and PsA in ILAR JIA classification does not correspond to the traditional concept of SpA because such ILAR criteria precludes the overlapping of inclusion criteria among different subgroups. Thus, the features that link ERA and PsA as SpA are the ones that may be thought to make them incompatible with each other. In contrast, the concept of SpA behind the ASAS axial and peripheral SpA criteria [11,12] reflects the Moll and Wright’s [27] original idea of clinical overlaps between the different diseases. This author’s opinion is that we need common concepts and criteria if we want to keep juvenile and adult onset types of SpA as a disease continuum. Amor, et al. [37] and ESSG [38] criteria have been validated in children and at least the latter has been used to some extent (Table 5). Today, ASAS axial and peripheral SpA criteria might be a good substitute for Amor, et al. [37] and ESSG [38]criteria and perhaps a good alternative to ILAR criteria [29]. Regarding nomenclature, it seems more appropriate for pediatric rheumatologists to use of the ILAR terms ERA and PsA than the contradictory term “peripheral SpA” since most children and adolescents have peripheral disease and rarely axial symptoms.

2) If needed, which set of ASAS criteria is more appropriate for children, axial or peripheral? It seems clear that axial and peripheral SpA classifications have different purposes. While the former [11] is intended to identify the spinal and sacroiliac involvement in the early inflammatory stage of AS, the latter [12] relies on peripheral arthritis, enthesitis, and dactylitis as entry criteria (Table 3).Regarding axial involvement, children and adolescents may have both active sacroiliitis on MRI (Figure 3) and radiographic sacroiliitis grade 2 bilateral or grades 2 or 4 unilateral (Figure 4), but in most cases these events occur in association with peripheral arthritis and enthesitis (Figure 5).Axial symptoms, as isolated features, are unusual in youngsters. The ASAS axial SpA criteria suggest the need for a history of back pain for at least three months as entry criteria before performing MRI and/or radiographic studies of the sacroiliac joints. There seems to be no clear clinical rationale to perform MRI studies of the sacroiliac joints and the spine in children in the absence of back pain. Certainly, the logical criteria for children and adolescents is the ASAS peripheral SpA criteria since they include the most important signs and symptoms in patients with juvenile-onset SpA.Except for “good response to NSAIDs”, on that no specific reports in children exist, children and adolescents with juvenile-onset SpA could well fulfill all axial and peripheral ASAS SpA criteria (Table 6). The diagnostic properties of some of these criteria were determined during the validation of the Amor et al. [37] and ESSG [38] classification criteria of SpA [74] and in a comparative study of juvenile-onset AS and u-SpA with JRA [60]. As expected, the sensitivity of back pain in the validation study of SpA according ESSG [38] was very low, but its specificity very high (Table 5). In the latter study, sensitivity, specificity, and + LR of tarsitis and enthesopathy were very high suggesting that tarsitis should be considered an additional criterion in any classification criteria (Figure 6). The frequency of each criterion depends on the classification category. By definition, for example, IBP and radiographic sacroiliitis should be found in all patients with AS, whereas arthritis or enthesitis should be found in all patients with ERA. On the other hand, the definition of each criterion and its diagnostic value should be assessed in children. The question of whether ASAS criteria for axial and peripheral SpA [11,12] have any role in the classification of children with SpA, ERA, PsA, and even undifferentiated arthritis remains to be determined. Ideally, all related clinical conditions in children and adults should be encompassed under the same criteria in order to facilitate scientific communication and patients transition from childhood to adulthood medical care. From the therapeutic point of view, there should be some advantages if the management of juvenile and adult onset forms could have the same opportunity to be treated in the early inflammatory stage of the disease.

**Table 5:** European Spondylarthropathy Study Group classification criteria and results of their validation in children

Inflammatory back pain *or* synovitis –symmetric or predominantly in the lower limbs *plus*	
one of the following	
·	Positive family history
·	Psoriasis
·	Inflammatory bowel disease
·	Urethritis, cervicitis, or acute diarrhea within one month before arthritis
·	Buttock pain alternating between right and left gluteal areas
·	Enthesopathy
·	Sacroiliitis

Validated in 361 adults with SpA and 455 controls, such criteria showed a sensitivity of 86.7% and specificity of 87.0% ref. [38].

Validated in 2,982 rheumatic children ref. [74], the diagnostic properties of those criteria were 78.7% for sensitivity, 92.2% for specificity, 58.8% and 96.8% for positive and negative predictive values, respectively, 85.3% for positive likelihood ratio, and 90.3 for diagnostic accuracy. Sensitivity of the criterion “inflammatory back pain” was only 9.1% in children.

**Table 6:** Estimated prevalence of each of the items listed in the Assessment of Spondyloarthritis International Society classification criteria for axial and peripheral spondyloarthritis in the different categories of juvenile onset disease in children and adolescents*

	Ankylosing spondylitis	Undifferentiated spondyloarthritis	Enthesitis related arthritis	Psoriatic spondyloarthritis	Psoriatic arthritis not related to spondyloarthritis
Sacroiliitis on MRI	+++	+	+	+	-
Sacroiliitis on X-rays	++++	-	+	+	-
Inflammatory back pain	++++	+	+	+	-
Arthritis	++++	++++	++++	++++	++++
Enthesitis (heel pain)	+++	+++	+++	++	-
Uveitis	++	+	+	+	-
Dactylitis	+	++	++	++	+++
Psoriasis	+	-	-	++++	++++
Crohn’s/colitis	+	-	-	+	-
Good response to NSAIDs	+++	NA	NA	NA	NA
Family history for SpA	++	+	+	++	-
HLA-B27	+++	++	++	++	-
Elevated C reactive protein	+++	+++	+++	+++	++
Previous infection	+	-	-	-	-

*Based on the literature and on the opinion of local experts in the care of these children.

The presence of such items excludes the undifferentiated spondyloarthritis and enthesitis related arthritis as diagnosis.

MRI = magnetic resonance imaging. NSAIDs = Non-Steroidal Antinflammatory Drugs. NA = data not available. SpA = Spondyloarthritis.

## Conclusions

The dilemma of how to apply adult AS and related SpA criteria to children has challenged pediatric rheumatologists for decades. The ILAR JIA criteria for PsA and ERA do not corresponds well with adult AS criteria. The new ASAS criteria offer rheumatologists a chance to reexamine how children with SpA, ERA, PsA, and undifferentiated arthritis can fit and not fit into these new criteria. Adult and pediatric rheumatologists want to diagnose these patients described by the ASAS criteria early and be able to offer these patients aggressive TNF inhibitor therapy, when possible and affordable, to possibly prevent joint and bone damage.

The application of those specific strategies in children and adolescents with SpA is challenging as the most important manifestation in the early stage of disease is not inflammatory back pain as it is in adults, but peripheral arthritis and enthesitis. In this instance, the best approach to juvenile onset SpA according to ASAS criteria may be not to use the axial criteria but rather to use the peripheral set of criteria. The question of whether pediatric rheumatology needs new separate criteria for SpA, ERA, PsA, and undifferentiated arthritis remains controversial and goes against our need to encompass such similar adult and pediatric diseases under the umbrella of one set of criteria.

## Competing interests

The author declares that he has no competing interests.

## Author contribution

RB-V is the author and corresponding author and has designed and written the article. As author read and approved the final manuscript.
